# A Study on the Effect of Temperature on PAM-Improved Shield Tunneling Sandy Slurry

**DOI:** 10.3390/ma19132765

**Published:** 2026-06-30

**Authors:** Di Wang, Shufang Zhai, Kang Li

**Affiliations:** 1 China Institute for Geo-Environmental Monitoring, Beijing 100081, China; wangdi01@mail.cgs.gov.cn; 2 College of Civil Engineering, Henan University of Technology, Zhengzhou 450001, China; 15936043804@163.com

**Keywords:** shield tunneling, sandy muck, Polyacrylamide(PAM), molecular dynamics, temperature, image analysis

## Abstract

Polyacrylamide (PAM) is widely used to improve sandy muck in shield tunneling due to its excellent physicochemical properties. During shield excavation, the temperature of excavated soil varies with geological depth and equipment heat transfer, making it necessary to investigate the temperature effect on the performance of PAM-modified sandy muck. In this study, molecular dynamics (MD) simulations are employed to construct a (PAM, H_2_O)/α-SiO_2_ interfacial model. The microstructural evolution and interfacial interaction characteristics between PAM molecules and the α-SiO_2_ substrate are analyzed at the nanoscale under different temperature conditions. A structure–performance–mechanism relationship is established, forming a conceptual framework of the “configuration–interaction energy–stability” mechanism for PAM-modified sandy muck. The main findings are as follows: (1) The PAM exhibits the most stable interfacial bonding with α-SiO_2_ between 278 K and 318 K, primarily governed by electrostatic attraction and hydrogen-bond synergy. (2) Within this temperature range, PAM forms a dense and stable interfacial adsorption structure, whereas both thermodynamic stability and structural integrity decline outside it. (3) At 318K, the PAM/α-SiO_2_ system shows the most favorable hydrogen-bonding behavior, with orderly alignment of PAM and H_2_O molecules and optimal chain flexibility and adhesion capacity. Therefore, 318 K is the upper temperature limit reference point at which the improvement effect of PAM remains the most stable, providing theoretical guidance for temperature-controlled soil conditioning in shield tunneling.

## 1. Introduction

The earth pressure balance shield tunneling method has become one of the major technologies in underground tunnel construction. Widely distributed sandy strata, characterized by poor particle gradation, high permeability, and loose structure, often lead to engineering problems such as insufficient excavation face stability, mud cake formation on the cutterhead, and difficulties in muck transportation, thereby seriously affecting tunneling efficiency and construction safety [1,2]. To effectively control the mechanical and rheological properties of sandy muck, polymer-based materials have attracted increasing attention in recent years as environmentally friendly soil-conditioning agents. Among them, Polyacrylamide (Polyacrylamide, PAM) has been widely applied in shield muck conditioning due to its excellent water solubility, gel-forming ability, and interfacial adsorption capacity [3]. Guo et al. [4] used PAM to condition coarse sand samples with a water content of 10%, and found that when the concentration of PAM polymer solution was 0.3% and the injection ratio was 20–25%, the permeability of coarse sand could be effectively reduced. Numerous studies have verified the engineering application effectiveness of PAM in sandy soil modification [5,6,7,8,9,10,11,12,13,14,15].

As a polymer material, the properties of PAM are highly affected by temperature. Yang investigated the laminar yield stress of PAM under different temperature conditions and found that the yield stress decreased significantly with increasing temperature, indicating that high temperature weakens the structural stability and flow resistance of PAM [16]. Touré conducted rheological tests on PAM at elevated temperatures and demonstrated that increasing temperature significantly weakened the network structure of polymer molecules, resulting in reduced viscosity and structural stability [17]. Moradi-Araghi pointed out that PAM is prone to thermal hydrolysis of amide groups at temperatures above 90 °C, leading to volume shrinkage and performance deterioration, indicating that thermal disturbance plays a decisive role in the interfacial stability of polymer structures [18]. Uranta carried out rheological experiments at 90 °C and found that the hydrolysis rate of PAM molecules reached 75%, accompanied by a significant decrease in viscosity, further confirming that high temperature induces thermal degradation of PAM amide groups and results in performance failure [19].

In the actual shield tunneling environment, the temperature conditions of muck are often uncontrollable and may vary significantly due to factors such as geological burial depth, equipment heat transfer, and external hydrothermal coupling, which can greatly affect the conditioning performance of PAM. Therefore, it is necessary to investigate the influence of temperature on the performance and interaction mechanism of PAM-conditioned sandy muck, and to reveal the microstructural evolution and interfacial interaction characteristics of PAM molecules under different temperature fields. In recent years, molecular dynamics (MD) numerical simulation has been widely used to study the interaction between polymer materials and mineral particles. Based on our previous research on the mechanism of PAM-modified sandy muck using the MD method [20], this study further considers the temperature variation during muck improvement in practical engineering and the thermosensitive property of polyacrylamide (PAM), and constructs the (PAM, H_2_O)/α-SiO_2_ interface model using Materials Studio 2020 software. The COMPASSII force field was employed to perform dynamic simulations within the temperature range of 258–338 K. Key parameters, including binding energy, hydrogen bond number, radial distribution function, relative concentration distribution, and mean square displacement, were quantitatively analyzed. In this simulation, periodic boundary conditions are adopted to eliminate the influence of system boundary effects on the shear results. The error between the internal friction angle of the sand sample obtained from the simulation and the result of the laboratory test is controlled within 5%. From the nanoscale perspective, the evolution of PAM molecular adsorption configurations and interfacial interaction mechanisms under different temperature conditions was investigated, and the effect of temperature on the adsorption behavior of PAM on the α-SiO_2_ surface, which provides a molecular-level theoretical basis for understanding the influence of temperature on the conditioning effect of shield tunneling sandy soil.

## 2. Molecular Dynamics Model Construction

Revealing the interfacial interaction mechanism at the molecular scale is essential for understanding the conditioning mechanism of PAM e-conditioned sandy muck. Numerous studies have demonstrated the effectiveness of Molecular Dynamics (MD) simulation in characterizing material properties and predicting mechanical responses [21,22,23,24,25,26,27]. In this study, α-SiO_2_ crystal cells were used as the substrate to construct (PAM, H_2_O)/α-SiO_2_ interfacial models at different temperatures (258 K, 278 K, 298 K, 318 K, and 338 K). First, the initial α-SiO_2_ crystal cell was extracted from the crystal database, with lattice parameters a, b, c, α, β, and γ of 4.913 Å, 4.913 Å, 5.4052 Å, 90°, 90°, and 120°, respectively. Using the Cleave Surface function in the Build Surfaces module, the crystal was cleaved along the [0 0 1] plane with a thickness of three crystal cells. After orthogonalization and hydroxyl saturation treatment [28], the thickness of the upper and lower vacuum layers was set to 0 Å, and a 3 × 5 × 1 supercell was constructed using the supercell function. Following geometry optimization in the Forcite module, the stabilized lattice parameters a, b, c, α, β, and γ were 24.565 Å, 25.528 Å, 17.701 Å, 90°, 90°, and 90°, respectively.

The molecular structural formula of PAM is (C_3_H_5_NO)n, and its density was set to 1.005 g/cm^3^. Previous studies have shown that the number of water molecules does not affect the overall simulation trends, and that the interfacial interaction of polymer molecules remains unchanged when the degree of polymerization of acrylamide monomers reaches 50 [29,30,31]. Therefore, in this study, the degree of polymerization was set to 50, with one polymer molecular chain and 200 water molecules included in the model. The amorphous polymer structure was constructed using the Calculation function in the Amorphous Cell module, followed by the geometry optimization procedure described above. To better simulate actual conditions, the polymer model was constructed in the form of a constrained layer during the optimization process. The final stabilized lattice parameters a, b, c, α, β, and γ were 25.000 Å, 25.000 Å, 18.925 Å, 90°, 90°, and 90°, respectively.

**Figure 1:**
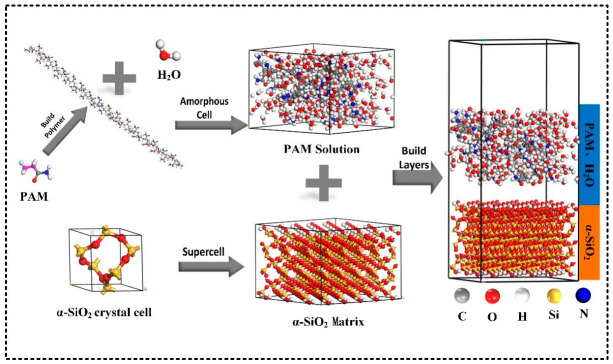
The specific process of creating a (PAM, H_2_O)/α-SiO_2_ model.

The final interfacial system model was constructed using the Build Layers module, as shown in Figure 1. A 2 Å vacuum layer was added above the α-SiO_2_ interface, and an additional vacuum layer with a thickness of 15 Å was introduced at the top of the entire system model to eliminate the influence of periodic boundary conditions on the surface layer [32]. The geometry optimization procedure described above was then further performed. After energy stabilization, the lattice parameters a, b, c, α, β, and γ were 24.782 Å, 25.264 Å, 57.349 Å, 90°, 90°, and 90°, respectively.

**Figure 2:**
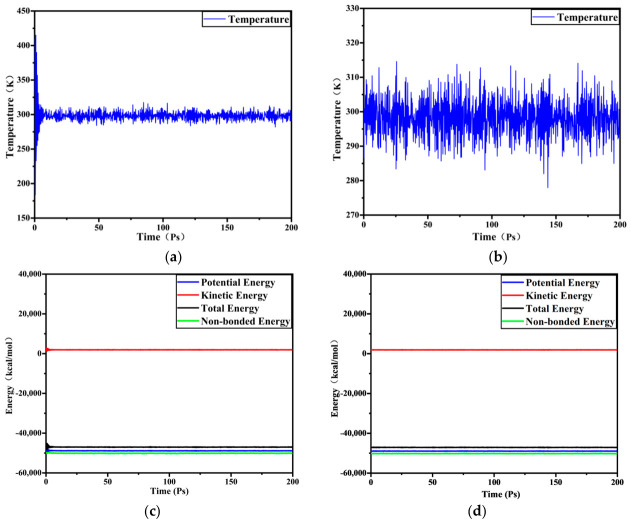
Temperature and energy–time curves at 298 K. (**a**) Temperature-time curve in NPT ensemble; (**b**) temperature-time curve in NVT ensemble; (**c**) energy–time curve in NPT ensemble; (**d**) energy–time curve in NVT ensemble.

During the model construction process, the COMPASSII force field with built-in charge parameters was employed throughout this study. The built-in charge parameters can accurately describe the structural stability, interfacial energy, and molecular interactions of organic–inorganic interfacial systems [33]. After model construction and energy minimization to a stable state, all system models in this study were simulated under both constant-pressure constant-temperature (NPT) and constant-volume constant-temperature (NVT) ensembles using the Dynamics module in Forcite. This procedure further relaxed the system under constant-volume conditions, enabling the model to approach a realistic stable state. The simulation time was set to 200 ps with a total of 200,000 steps, and one frame was output every 1000 steps. As shown in Figure 2, at 298 K, the temperature fluctuation gradually stabilized within 200 ps, while the energy variation remained stable throughout the simulation process, indicating that the constructed model possessed good structural stability and thermodynamic stability.

## 3. Results and Analysis

### 3.1. Binding Energy

Binding energy is an important parameter for characterizing interfacial interactions and adsorption strength [34]. A larger absolute value of binding energy indicates stronger interactions between PAM molecules and the α-SiO_2_ surface, corresponding to higher system stability. The calculation formula is as follows:

(1) $$E_{\mathrm{binding}}=E_{\mathrm{total}}-\left(E_{\mathrm{PAM}}+E_{\alpha \text{-}{SiO}_{2}}\right)$$

Table 1 and Table 2 present the binding energy and non-bond energy of the interfacial models at different temperatures after NPT and NVT equilibration under stable system conditions (100th frame).

From the results in the tables, the following conclusions can be drawn:

- The binding energy remains negative over the entire temperature range, indicating that the system is thermodynamically stable under all conditions, and there is an obvious attractive interaction between PAM molecules and the α-SiO_2_ surface;
- The absolute value of binding energy first increases and then decreases with increasing temperature, indicating that the interfacial interaction strength first strengthens and then weakens;
- In the normal temperature range (278–318 K), the binding energy of the (PAM, H_2_O)/α-SiO_2_ system is equal to the non-bond energy, which indicates that the interaction between PAM and α-SiO_2_ belongs to physical adsorption rather than chemical reaction, and all interactions originate from non-covalent bonds (electrostatic force and hydrogen bond). The silanol groups (Si-OH) on the quartz surface exist in three forms in an aqueous environment [35]: >SiOH_2_+ (positively charged, pKa < −2.5), >SiOH0 (neutral) and >SiO^−^ (negatively charged, pKa = 4.8–9.3), > denotes bulk quartz. Under near-neutral conditions, the quartz surface is dominated by negatively charged SiO^−^. The partially positively charged -NH_2_ groups in PAM molecules generate electrostatic attraction with SiO^−^. Meanwhile, the polar groups -NH_2_ and -C=O of PAM form a stable adsorption structure with the surface Si-OH through hydrogen bonds. A hydrogen bond is the non-covalent interaction between the polar groups (-NH_2_ or -C=O) of PAM and the -OH (hydroxyl) groups on the α-SiO_2_ surface. Coulomb electrostatic interaction dominates in the non-bond energy, indicating that due to the polarity of PAM molecules, PAM molecules in solution mainly interact with α-SiO_2_ through electrostatic attraction.
- Coulomb electrostatic interaction dominates the non-bond energy, suggesting that PAM molecules interact with α-SiO_2_ mainly through electrostatic attraction due to their polar nature.

In addition, it can be observed that:

1. At 258 K, the binding energy between PAM molecules and the α-SiO_2_ surface is the lowest and not equal to the non-bond energy. This is mainly because at low temperatures, fewer hydrogen bonds exist between water molecules, making it difficult for PAM molecules to stably attach to the α-SiO_2_ surface, and hydrogen bond formation is restricted.
2. At 318 K, the interaction between PAM molecules and α-SiO_2_ is the strongest. This is attributed to an optimized hydrogen-bond network between PAM chains and water molecules, which promotes stable adsorption of PAM molecular chains.
3. At 338 K, thermal motion of PAM molecules increases, disturbing the hydrogen-bond network and gradually reducing the number of hydrogen bonds.

Based on the binding energy analysis, these variations reveal the adsorption behavior of PAM under different temperatures. The PAM exhibits the most stable interaction with the α-SiO_2_ interface within 278–318 K, mainly governed by the synergistic effects of electrostatic interaction and hydrogen bonding. At the macroscopic scale, this corresponds to stable performance and better conditioning effectiveness of PAM-treated sandy soil within this temperature range, while extremely low or high temperatures weaken adsorption capacity and are unfavorable for macroscopic conditioning performance.

### 3.2. Hydrogen Bond Statistics

**Figure 3:**
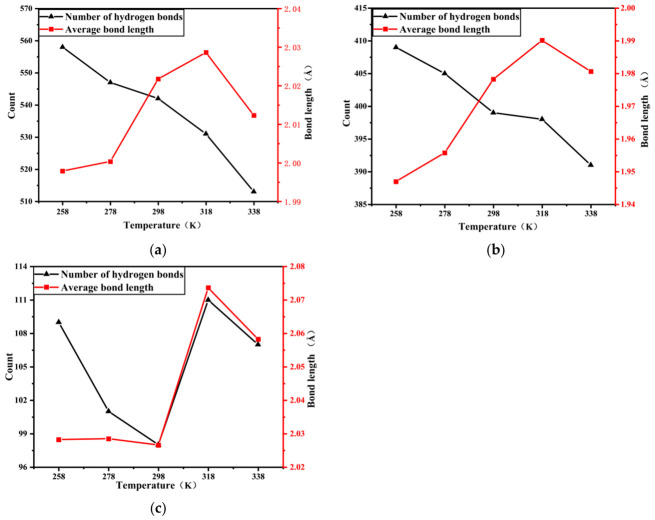
The number and bond length of hydrogen bonds in the system at different temperatures. (**a**) Whole (PAM, H_2_O)/α-SiO_2_ system; (**b**) local H_2_O/α-SiO_2_ system; (**c**) local PAM/α-SiO_2_ system.

Hydrogen bonding plays an important role in analyzing the interfacial interactions between PAM molecules and the α-SiO_2_ surface [36]. To investigate the effect of temperature on intermolecular hydrogen-bonding behavior in the system, Figure 3a–c present the number of hydrogen bonds and hydrogen-bond lengths for the overall (PAM, H_2_O)/α-SiO_2_ system, the local H_2_O/α-SiO_2_ system, and the local PAM/α-SiO_2_ system at different temperatures. The results show that:

(1) For the overall (PAM, H_2_O)/α-SiO_2_ system, the number of hydrogen bonds decreases with increasing temperature, while the average hydrogen-bond length exhibits a typical trend of first increasing and then decreasing. This indicates that increasing temperature weakens the stability of the hydrogen-bond network between PAM molecules and water molecules. This trend is consistent with the decrease in binding energy, suggesting that hydrogen-bond rupture is one of the main factors leading to the reduction of interfacial binding strength.

(2) For the local H_2_O/α-SiO_2_ system, both the number of hydrogen bonds and the average hydrogen-bond length exhibit a temperature-dependent trend similar to that of the overall system. This is because water molecules are more susceptible to thermal disturbance at higher temperatures, resulting in reduced adsorption capacity on the silicate surface and weakened hydrogen-bond stability at the water–substrate interface.

(3) For the local PAM/α-SiO_2_ system, the number of hydrogen bonds shows a trend of first decreasing and then increasing, indicating that at moderate temperatures, PAM chains can adsorb onto the α-SiO_2_ surface in a more stable configuration with the mediation of water molecules. Meanwhile, the hydrogen-bond length shows a non-monotonic trend with decreasing–increasing–decreasing behavior, indicating a balance between configurational adjustment and thermal disturbance.

Hydrogen bond analysis shows that increasing temperature significantly weakens the number and strength of the hydrogen-bond network in the system. However, at a moderate temperature (around 318 K), the PAM/α-SiO_2_ system still exhibits relatively favorable hydrogen-bond adsorption behavior, which is in good agreement with the variation trend of binding energy. This indicates that interfacial interactions are relatively strong under this temperature condition. At the macroscopic scale, the sandy muck conditioned by PAM under this temperature condition exhibits a better slump value and improved flow plasticity.

### 3.3. Radial Distribution Function

The radial distribution function can quantitatively characterize the local distribution characteristics between particles and the structural ordering of the local system [37]. The calculation formula is as follows:

(2) $$g\left(r\right)=\frac{dN}{4\pi r^{2}pdr}$$

where r represents the distance between particles, N represents the number of particles in the system, and ρ represents the average density of the system.

**Figure 4:**
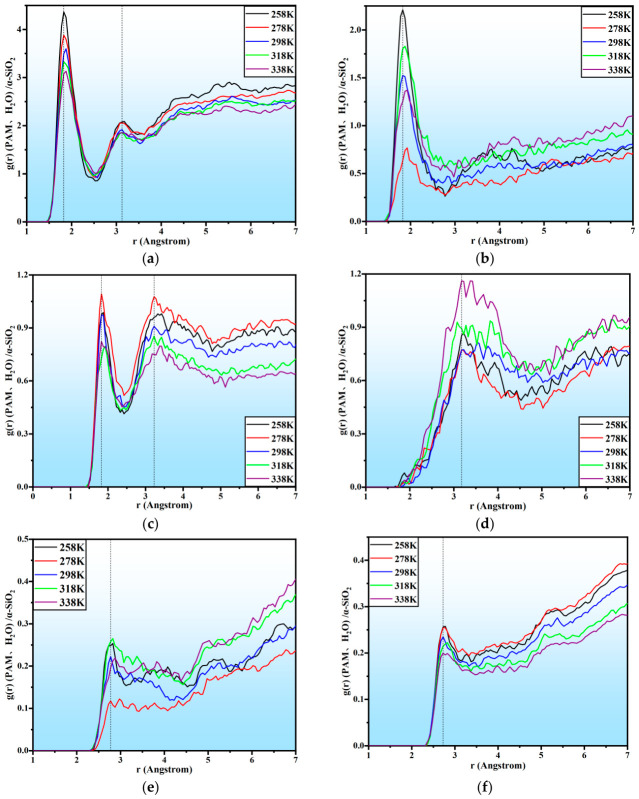
Radial distribution functions of each atom at different temperatures. (**a**) O(PAM)-H(H_2_O); (**b**) H(α-SiO_2_)-O(PAM); (**c**) H(α-SiO_2_)-O(H_2_O); (**d**) H(α-SiO_2_)-N(PAM); (**e**) O(PAM)-O(α-SiO_2_); (**f**) O(H_2_O)-O(α-SiO_2_).

Figure 4a–f present the radial distribution functions at different temperatures between H atoms in α-SiO_2_ and O atoms in H_2_O, O atoms in PAM, and N atoms in PAM; between O atoms in PAM and H atoms in H_2_O as well as O atoms in α-SiO_2_; and between O atoms in H_2_O and O atoms in α-SiO_2_.; as well as between O atoms in H_2_O and O atoms in α-SiO_2_. The position of the first main peak in the radial distribution function reflects the most probable average distance between two specific atoms in the system, while the peak intensity represents the interaction strength between them.

Distinct characteristic peaks appear at 1.825 Å (the characteristic hydrogen-bond distance) in Figure 4a–c, indicating the existence of typical hydrogen bonds between the three groups of atomic pairs.

(1) In Figure 4a, the peak intensity gradually decreases with increasing temperature, indicating that stronger interactions between water molecules and PAM chains are maintained under low and moderate temperature conditions, which is favorable for the formation of a stable hydrogen-bond network between PAM amide groups and water molecules.

(2) In Figure 4c, the peak intensity reaches its maximum at 278 K, indicating that water molecules can stably adsorb onto the α-SiO_2_ interface under relatively low-temperature conditions. This also suggests that water molecules act as intermediates to enhance the stability of the overall adsorption structure within the low-temperature range.

(3) In Figure 4b, the peak intensity is the highest at 258 K and the lowest at 278 K, indicating that although PAM exhibits strong adsorption capacity at low temperature, its structural ordering is relatively poor, and the adsorption behavior is dominated by static aggregation, making it difficult to maintain a stable interfacial structure. The increase in peak intensity at 298 K suggests that the adsorption ordering is improved under moderate temperature conditions, indicating an optimal balance between molecular configuration and adsorption stability.

Distinct secondary peaks appear at 2.725 Å (corresponding to medium-range polar adsorption or coordination interactions) in Figure 4e,f.

(1) In Figure 4e, the main peak reaches its maximum at 318 K, indicating that PAM molecular chains exhibit the most extended configuration at this temperature, forming medium-range polar coordination interactions with the α-SiO_2_ interface. This reflects the optimal temperature range for PAM/α-SiO_2_ adsorption and further confirms the synergistic enhancement between binding energy and hydrogen bonding.

(2) In Figure 4f, the peak intensity reaches its maximum at 278 K, indicating that a stable polar wetting film can still form between H_2_O and α-SiO_2_ under relatively low-temperature conditions. However, with increasing temperature, the adsorption strength decreases significantly, indicating intensified desorption of water molecules and gradual disruption of the lubrication network.

Distinct characteristic peaks appear at 3.125 Å (corresponding to long-range weak interactions or non-directional adsorption characteristics) in Figure 4a,c,d, indicating the presence of distinct secondary peaks.

(1) These results indicate that non-directional van der Waals interactions or diffusive contacts exist among the three groups of atomic pairs at elevated temperatures.

(2) In Figure 4a, the peak intensity at 338 K increases slightly, indicating that water molecules desorb from PAM chains and enter a free diffusion state.

(3) In Figure 4c, the peak remains at 1.825 Å throughout the entire temperature range, indicating that stable short-range hydrogen-bond interactions between H_2_O and α-SiO_2_ are maintained and are not easily disturbed by thermal motion.

(4) In Figure 4d, the peak intensity increases with increasing temperature. This trend indicates that elevated temperature significantly affects the molecular configuration of PAM. Enhanced thermal motion of molecular chains causes polar groups (NH_2_) that were originally folded or buried inside the chains to migrate more easily toward the interface, thereby participating in the adsorption process.

Based on the radial distribution function analysis, it can be concluded that a relatively dense and stable adsorption structure is formed between PAM and the α-SiO_2_ interface within the temperature range of 278–318 K. In contrast, the interfacial interactions are weakened at 258 K and 338 K, accompanied by the simultaneous deterioration of thermodynamic and structural characteristics. These results indicate that temperature significantly affects the adsorption behavior between PAM and the α-SiO_2_ interface. Within the temperature range of 278–318 K, PAM exhibits relatively stable performance in sandy soil conditioning.

### 3.4. Relative Concentration Distribution

The relative concentration distribution curve is used to describe the spatial distribution of specific types of atoms or molecules and the relative density distribution within the system [38,39,40]. The calculation formula is as follows:

(3) $$\mathrm{Relative}\left[\mathrm{set}\right]=\frac{{\left[\mathrm{set}\right]}_{\mathrm{slab}}}{{\left[\mathrm{set}\right]}_{\mathrm{bulk}}}.$$

where Relative[set] represents the relative concentration of the corresponding region, ${\left[\mathrm{set}\right]}_{\mathrm{slab}}$ represents the concentration in the local region, and ${\left[\mathrm{set}\right]}_{\mathrm{bulk}}$ represents the concentration in the entire unit cell structure.

**Figure 5:**
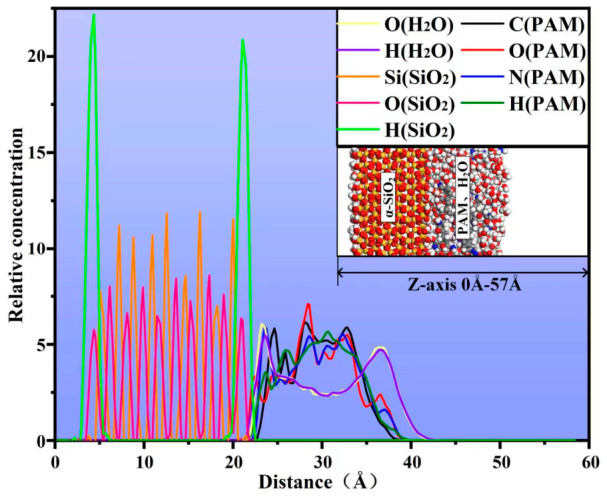
The relative concentrations of each atom in the system at 298 K.

Figure 5 presents the relative concentration distribution of atoms in the system at 298 K. The results show that:

(1) Near the α-SiO_2_ surface (0–22 Å), distinct concentration peaks of Si atoms, O atoms, and hydroxylated H atoms are observed, indicating the high structural stability of the α-SiO_2_ substrate.

(2) The atoms in the PAM and α-SiO_2_ exhibit an ordered arrangement without overlapping characteristic peaks, indicating that a certain degree of interfacial separation is maintained between PAM and α-SiO_2_. This phenomenon may be attributed to nanoscale gaps or boundary effects.

(3) The H and O atoms in H_2_O molecules are mainly distributed within the range of 22–43 Å, and their peak intensities are generally lower than those associated with PAM molecules. This indicates that PAM molecules and H_2_O molecules initially compete for adsorption sites, after which PAM molecules occupy more adsorption sites on the α-SiO_2_ surface.

(4) The characteristic peaks of C, O, N, and H atoms in PAM (22–40 Å) all appear after those of α-SiO_2_ atoms, and significant overlap of the distribution curves occurs near 23–27 Å. This indicates that PAM molecules undergo chain folding and aggregation when approaching the α-SiO_2_ surface, mainly due to electrostatic attraction and hydrogen-bond interactions.

**Figure 6:**
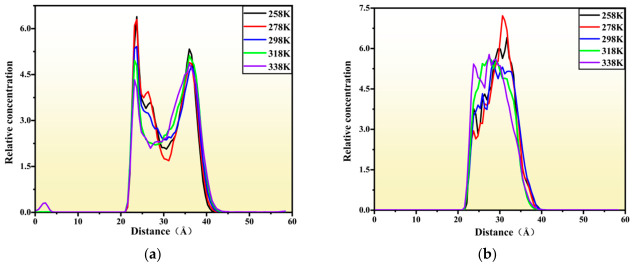
The relative concentration distribution of H_2_O and PAM in the local system under different temperature. (**a**) Relative concentration of H_2_O in the system at different temperatures; (**b**) relative concentration of PAM in the system at different temperatures.

Figure 6 presents the relative concentration distributions of H_2_O and PAM in the local system under different temperature conditions. The results show that:

(1) The main peak of the relative concentration distribution of H_2_O molecules gradually decreases with increasing temperature, indicating that the ordering of water molecules near the interface is weakened at elevated temperatures, leading to weakened adsorption capacity. Some water molecules desorb from the α-SiO_2_ surface due to thermal disturbance.

(2) The main peak of the relative concentration distribution of PAM molecules gradually changes from a concentrated state to a more uniform distribution with increasing temperature. At 318 K, the widest adsorption band is formed, indicating that PAM molecular configurations are more uniformly distributed at the interface and that the molecular chains achieve the highest degree of extension, which is beneficial for the formation of a stable adsorption structure. In contrast, at 278 K, PAM exhibits a sharp peak, reflecting significant local molecular aggregation and non-uniform interfacial distribution, which is consistent with the radial distribution function results.

Based on the relative concentration distribution analysis, it can be concluded that at 318 K, PAM molecules exhibit a relatively ordered arrangement and H_2_O molecules exhibit a more uniform distribution, resulting in stronger interfacial adsorption. This indicates that this temperature is more favorable for the formation of a stable adsorption structure in the PAM/α-SiO_2_ interfacial system and is beneficial for the conditioning performance of PAM for sandy soil.

### 3.5. Mean Square Displacement

Mean square displacement (MSD) is an important statistical parameter for evaluating the dynamic motion behavior of atoms or molecules over time. It can directly reflect the diffusion behavior of particles and provide insight into their adsorption–desorption processes [41]. A higher MSD value indicates faster atomic motion and lower system stability. The calculation formula is as follows:

(4) $$\mathrm{MSD}={\left.\left|r\left(t\right)-r\left(0\right)\right.\right|}^{2}$$

where r(t) represents the position of a particle at time t, and r(0) represents the initial position of the particle.

**Figure 7:**
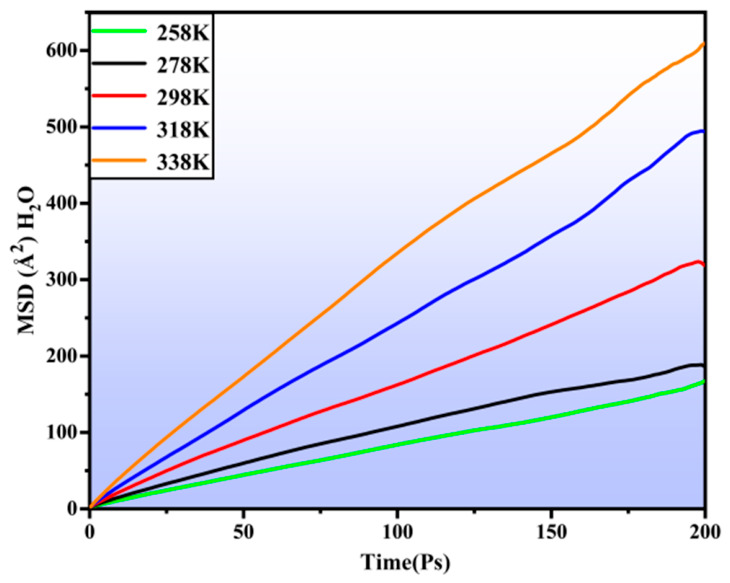
The average displacement of H_2_O in the system at different temperatures.

Figure 7 presents the mean square displacement (MSD) curves of H_2_O molecules in the system at different temperatures. It can be observed that the MSD of H_2_O molecules increases with increasing temperature, indicating that the diffusivity of water molecules gradually increases. The MSD of water molecules reaches its maximum at 338 K and its minimum at 258 K, indicating that the thermal motion of water molecules is intensified and interfacial adsorption capacity is weakened at elevated temperatures. In contrast, the MSD of water molecules at 318 K remains within a moderate range, indicating that water molecules can still maintain a certain degree of an ordered interfacial structure at this temperature. Meanwhile, the relatively high diffusivity of water molecules enables them to wet the α-SiO_2_ substrate surface at the interface, thereby providing important dynamic support for the interfacial adsorption of PAM molecules.

**Figure 8:**
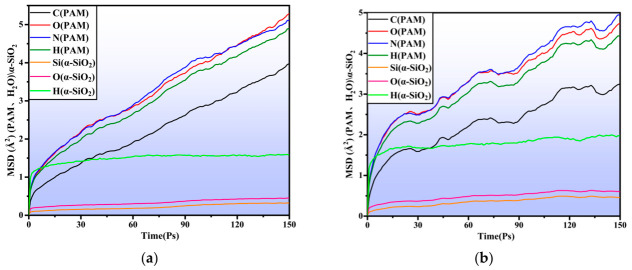

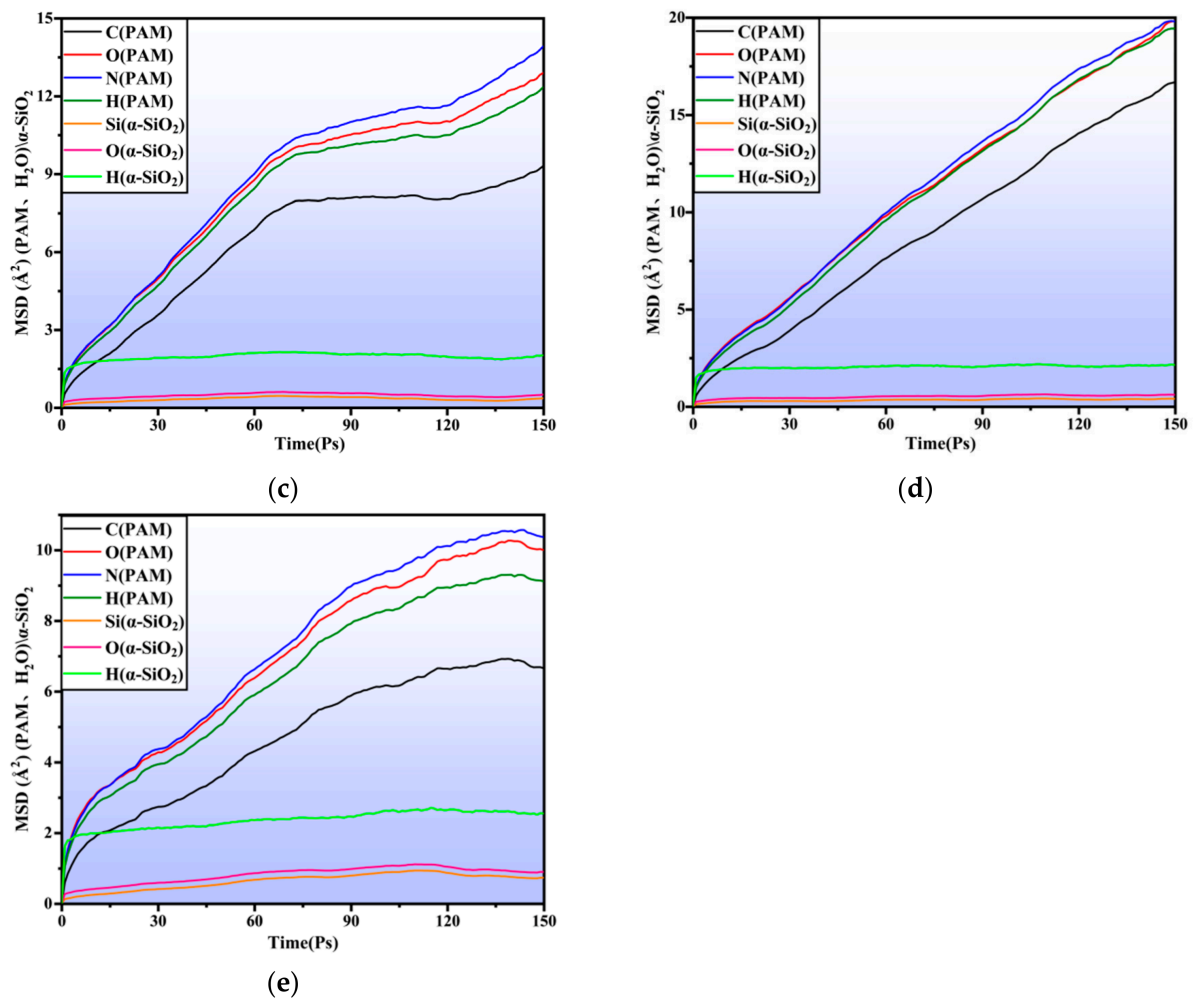
The displacement diagrams of each atom of PAM molecules and the α-SiO_2_ matrix at different temperatures. (**a**) Mean square displacement-time curve of atoms at 258 K; (**b**) mean square displacement-time curve of atoms at 278 K; (**c**) mean square displacement-time curve of atoms at 298 K; (**d**) mean square displacement-time curve of atoms at 318 K; (**e**) mean square displacement-time curve of atoms at 338 K.

Figure 8 presents the mean square displacement (MSD) of all atoms in PAM molecules and the α-SiO_2_ substrate at different temperatures. The following observations can be made from Figure 8a–e:

(1) Across all temperature ranges, the MSD values of O, H, and Si atoms in α-SiO_2_ remain the lowest among all atoms, indicating that the α-SiO_2_ substrate structure is not significantly affected by thermal motion or disturbances from other particles in the system. This also confirms the suitability of α-SiO_2_ as a rigid substrate.

(2) The MSD of PAM molecules exhibits an initial increase followed by a decrease under different temperature conditions. Within the temperature range of 258–318 K, the MSD values of PAM atoms gradually increase with increasing temperature, indicating that thermal motion enhances the mobility of molecular chains. At 318 K, PAM molecular chains exhibit the highest mobility, and thermal motion drives rapid chain extension, resulting in the highest MSD values and the most favorable chain extension state. At 338 K, the MSD decreases significantly, indicating that elevated temperature weakens hydrogen bonding and electrostatic adsorption forces, leading to desorption, local chain collapse, or restricted molecular motion of PAM molecules, thereby reducing adsorption stability.

Based on the mean square displacement (MSD) analysis, it can be concluded that within the temperature range of 258–298 K, the enhanced mobility of PAM molecules is beneficial for the formation of a stable adsorption structure. At 318 K, PAM molecular chains exhibit relatively strong chain flexibility and interfacial adhesion ability, and the adsorption state of the system remains relatively stable without obvious desorption or severe thermal disturbance. Therefore, this temperature is favorable for sandy soil conditioning using PAM.

### 3.6. Macroscopic Modification Tests at 298 K

The sandy soil used in this study was collected from a shield tunneling construction site. According to sieve analysis, the coefficient of uniformity (Cu) and coefficient of curvature (Cc) were determined to be 5.97 and 0.88, respectively, indicating that the soil is classified as poorly graded medium sand. The natural water content of the soil was 7.89%, the maximum dry density was 1.66 g/cm^3^, and the optimum water content was 9%. An anionic PAM supplied by a company in Zhengzhou, China, was used, with a molecular weight of 3.0 × 10^7^ g/mol.

**Figure 9:**
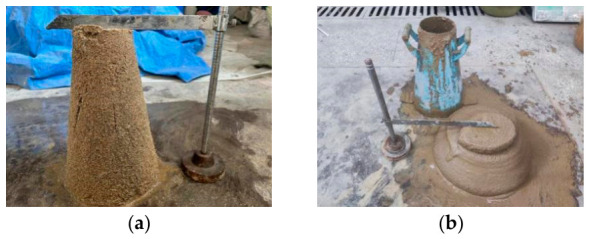
Slump change of shield muck before and after modification. (**a**) Before modification; (**b**) after modification.

In the macroscopic experiments, sandy soil specimens were prepared using PAM solution with a concentration of 0.5% and a mixing ratio of 25%, followed by slump tests, permeability tests, and direct shear tests. The slump test is conducted using a standard slump cone. After the sand collapses under its own weight, the stable slump value is measured, so as to analyze the influence of PAM on the fluidity of sand. As shown in Figure 9a,b, the conditioned sandy muck exhibited good flowability and water-retention performance.

The constant-head permeability test was conducted using a TST-70 constant-head permeameter to measure the permeability coefficient of sand before and after modification with PAM; the direct shear test was carried out with a ZJ strain-controlled direct shear apparatus. The specimen is 61.8 mm in diameter, 20 mm in height, with a set shear rate of 1.2 mm/min and a normal force ranging from 100 kPa to 400 kPa. By measuring the shear strength at failure, the cohesion c and internal friction angle φ of the sand are calculated based on Coulomb’s law, and then the influence of PAM on the change of macroscopic properties of sandy muck is analyzed. Table 3 presents the results of permeability and direct shear tests of sandy muck before and after PAM treatment. The permeability coefficient decreases from 7.95 × 10^−4^ cm s^−1^ to 2.59 × 10^−4^ cm s^−1^ after conditioning, indicating that PAM effectively reduces the permeability of sandy muck. This is mainly attributed to PAM filling the pores between soil particles, reducing the number and size of flow channels, and significantly improving the anti-seepage performance of the soil.

After treatment, the cohesion increases from 3.18 kPa to 10.08 kPa, indicating that PAM forms a bonding layer between soil particles through its molecular chains, thereby enhancing interparticle cohesion. Meanwhile, the internal friction angle decreases from 41.49° to 36.83°, suggesting that PAM reduces friction between soil particles.

The results indicate that PAM treatment of sandy muck exhibits a dual effect on its mechanical properties. On the one hand, it increases soil cohesion, leading to enhanced interparticle bonding and a more tightly packed soil structure. On the other hand, it reduces the internal friction angle, thereby improving the deformation behavior of the soil.

### 3.7. Scanning Electron Microscopy (SEM) Observation at 298 K

Observing the surface morphology and interface connection of sand particles using the Guoyi Quantum SEM 5000X device. At 298 K, the surface morphology of sandy muck before and after PAM treatment was observed and analyzed using SEM. In this study, sandy muck samples before conditioning (water content of 9%, without PAM) and after conditioning (water content of 9%, PAM concentration of 0.5%, injection ratio of 25%) were selected for comparison.

**Figure 10:**
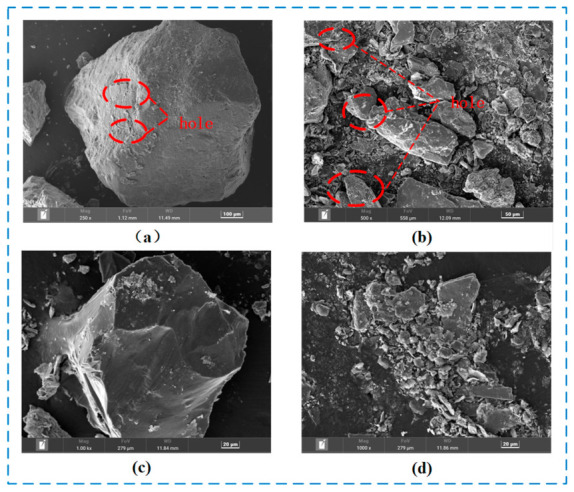
SEM images of different samples. (**a**) Before modification (250×); (**b**) before modification (500×); (**c**) after modification (1000×); (**d**) after modification (1000×).

The SEM images show that the untreated sandy muck exhibits a dispersed particle structure with a highly developed pore system. As shown in Figure 10a (250×), the particle surfaces are rough with evident micropores, resulting in a loose and porous structure. In Figure 10b (500×), obvious pores and layered structures can be observed on the particle surfaces, with sharp angular edges between particles, indicating a loosely packed interparticle arrangement. These microstructural features reflect the high porosity, weak interparticle bonding, and unstable natural structure of the sandy muck.

After conditioning with PAM, significant microstructural changes are observed in the sandy muck. In Figure 10c (1000×), the particle surfaces are coated with a continuous thin film, and the sharp edges are significantly blunted. The surface becomes smoother, and part of the micro-pores is filled, resulting in a denser overall structure. In Figure 10d (1000×), the particle surfaces appear smooth without obvious pore structures, and the particles are more closely packed. These observations indicate that PAM molecules coat the particle surfaces and effectively fill the interparticle voids.

SEM analysis indicates that at 298 K, PAM forms a flexible polymer film on the surface of soil particles, thereby reducing pore space and decreasing soil permeability. The presence of PAM enhances the packing density and overall structural compactness of the sandy muck, strengthens interparticle bonding, and improves the mechanical stability of the soil structure, leading to enhanced deformation resistance.

SEM analysis indicates that at 298 K, PAM forms a continuous polymer film on the surface of soil particles, thereby reducing pore space and decreasing soil permeability. The presence of PAM enhances the packing density and overall structural compactness of the sandy muck, strengthens interparticle bonding, and improves the mechanical stability of the soil structure, thus enhancing its deformation resistance.

## 4. Conclusions

Based on the multi-scale experimental and simulation results, the following conclusions can be drawn:

(1) Molecular dynamics (MD) simulations reveal that the interfacial interaction between PAM and α-SiO_2_ is highly temperature-dependent. Within the temperature range of 278–318 K, the system exhibits relatively stable binding energy, hydrogen-bond network, and interfacial structure, indicating enhanced adsorption performance under moderate temperature conditions.

(2) Radial distribution function (RDF) and hydrogen bond analyses indicate that characteristic hydrogen bonds are primarily located at 1.825 Å, while medium-range interactions are observed at 2.725 Å. These results confirm that electrostatic interactions and hydrogen bonding synergistically govern the adsorption behavior of PAM on the α-SiO_2_ surface.

(3) Mean square displacement (MSD) results indicate that molecular mobility increases with temperature, reaching an optimal balance at 318 K, where PAM chains exhibit enhanced configurational flexibility and interfacial stability. Excessively high temperatures weaken hydrogen bonding and reduce adsorption stability due to intensified molecular thermal motion.

(4) Relative concentration distribution analysis shows that at 318 K, PAM molecules exhibit a more uniform and ordered interfacial arrangement, while water molecules maintain a stable distribution, contributing to a more stable adsorption structure.

(5) SEM observations demonstrate that PAM forms a continuous polymer film on soil particle surfaces, significantly reducing pore space, increasing soil compactness, and enhancing interparticle bonding. This improves the permeability resistance and deformation stability of the treated sandy muck.

(6) Macroscopic tests demonstrate that PAM treatment significantly reduces permeability, increases cohesion, and decreases the internal friction angle. This indicates that PAM improves soil performance through a dual mechanism of strengthening particle bonding and modifying interparticle friction.

Overall, the optimal conditioning performance of PAM-treated sandy muck is achieved at 278–318 K, where interfacial interactions, molecular structure stability, and macroscopic mechanical properties are simultaneously optimized.

## Figures and Tables

**Table 1:** The binding energy of (PAM, H_2_O)/α-SiO_2_ at different temperatures.

Binding Energy	E_binding_	E_total_	E_PAM_	$E_{\alpha \text{-}{SiO}_{2}}$
258 K	−207	−49,333	−3626	−45,500
278 K	−201	−49,172	−3572	−45,399
298 K	−208	−49,078	−3527	−45,343
318 K	−223	−48,834	−3349	−45,262
338 K	−219	−48,700	−3262	−45,219

**Table 2:** The non-bonding energies of (PAM, H_2_O)/α-SiO_2_ at different temperatures.

Non-Bond Energy	E_non-bonding_	E_vdW_	E_LRC_	E_Coulomb_
258 K	−151	−51	−17	−82
278 K	−201	−50	−17	−134
298 K	−208	−46	−17	−145
318 K	−223	−57	−17	−149
338 K	−219	−72	−17	−130

**Table 3:** Test results of permeability test and direct shear test on muck before and after improvement.

Experimental Types		Before Conditioning	After Conditioning
Permeability test	Permeability coefficient (cm/s)	7.95 × 10^−4^	2.59 × 10^−4^
Direct shear test	Cohesion (kPa)	3.18	10.08
	Internal friction angle (°)	41.49	36.83

## Data Availability

The original contributions presented in this study are included in the article. Further inquiries can be directed to the corresponding author.
